# Compositional Changes in Foliage Phenolics with Plant Age, a Natural Experiment in Boreal Forests

**DOI:** 10.1007/s10886-017-0881-5

**Published:** 2017-08-29

**Authors:** Hilde Karine Wam, Caroline Stolter, Line Nybakken

**Affiliations:** 1 Division of Forest and Forest Resources, Norwegian Institute of Bioeconomy Research (NIBIO), 1431 Ås, Norway; 2 Department of Animal Ecology and Conservation, University of Hamburg, 20146 Hamburg, Germany; 3 Faculty of Environmental Sciences and Natural Resource Management, Norwegian University of Life Sciences, 1432 Ås, Norway

**Keywords:** Apparency, Assart, C: N ratio, Clearcut, Defence, Herbivory, Plant secondary metabolites, Plant: Animal

## Abstract

The composition of plant secondary metabolites (PSMs) extensively impacts ecosystem functioning. It is vital that we understand temporal patterns in the plants’ allocation of resources to PSMs, particularly those influenced by human activity. Existing data are insufficient in the long-term perspective of perennial plants (age or ontogeny). We analysed phenolic concentrations in foliage from birch (*Betula pubescens* Ehr*.*) considered to be undamaged and growing on 5, 10 and 15 years old clear-cuts in two boreal forest landscapes in Norway, sampled at the peak of the growing season. In sum, low molecular weight phenolic concentrations decreased with age. Apart from one apigenin glycoside, the low molecular weight phenolics co-varied similarly at all ages, suggesting a lack of temporal compound-specific prioritisation of this group. In contrast, the concentration of MeOH-soluble condensed tannins increased with age. The compositional shift fits well with several hypotheses that may provide proximate explanations for age patterns in PSM allocations, including both resource constraints and external pressures. Regardless of these explanations, our study adds an important perennial perspective (plant age) to temporal PSM patterns already well-known in boreal plant phenology (foliage age).

**Electronic supplementary material:**

The online version of this article (10.1007/s10886-017-0881-5) contains supplementary material, which is available to authorized users.

## Introduction

The composition of plant secondary metabolites (PSMs) extensively impacts ecosystem functioning (Hagerman and Robbins 1993; Kraus et al. 2003; Provenza et al. 2007). Given the current rate of alterations to land (Leal et al. 2014) and atmosphere (Lindroth 2012), we need to clarify how such changes may temporally affect plant allocation to PSMs. Temporal changes may be induced through modifications of, for example, light intensity (Hansen et al. 2006), nutrient access (Fjære et al. 2016; Koricheva et al. 1998), atmospheric gases (Couture et al. 2014), allelopathy (Mandal et al. 2010), weather (Lambers et al. 1998), fire (Lavoir et al. 2013), herbivory (Elger et al. 2009) or their interactions (Jarzomski et al. 2000).

Practically all meta-analyses of temporal PSM patterns have been made in a phenological context (i.e. within season development) (e.g. Aide 1993; Asch and Visser 2007; Koricheva and Barton 2012). A seasonal decrease in the total PSM concentration seems most prevalent across genotypes and species, but divergent trends are often observed between herbaceous and woody plants (Koricheva and Barton 2012). One proposed explanation is the fact that herbaceous plants have no dormant above-ground parts that require protection during the cool season. This highlights how ontogeny, by often spanning longer periods, is a different context than seasonal phenology. Empirical studies on PSM allocation in the long-term perspective of ontogeny (plant age) are scarce, and patterns remain inconsistent and unclear (Barton and Koricheva 2010). This is unfortunate, because the effects of most alterations of ecosystems extend beyond season.

There are two major paradigms on how PSMs evolve with plant (or tissue) age (Stamp 2003). The first one considers patterns to be the results of resource constraints, i.e. “bottom-up” hypotheses. These generally predict that PSM concentrations increase with plant age (Bryant et al. 1983; Herms and Mattson 1992; Loomis 1932). For example, the ‘protein competition’ model (Jones and Hartley 1999) uses plant metabolism to argue that protein and most phenolic syntheses compete for the common precursor phenylalanine. When nitrogen limitation increases (e.g. with forest succession), growth should be more restricted than phenolic synthesis, because only growth also requires other N-containing amino acids. The other paradigm centres on external pressures, i.e. “top-down” hypotheses, where much focus has previously been on herbivory risk (Feeny 1976; McKey 1974; Rhoades 1979; Rhoades and Cates 1976). However, plants are exposed to a large suite of risks, including abiotic factors such as photodamage (Close and McArthur 2002), frost and drought (Samanta et al. 2011). Herbivory in isolation quickly becomes a too simplistic focus to explain ontogenetic PSM patterns. New approaches are emerging that advocate comprehensive views that consider resource constraints and external pressures in a dynamic context (e.g. ‘the extended phenotype’, Bailey et al. 2012).

Because of the variety of life strategies of different plants, we are likely to find empirical support for several hypotheses explaining age patterns in PSM allocation. However, the data may also appear ambiguous for the same plant species in comparable settings. This may simply be a result of immature rather than unsuitable theory (Stamp 2003). It may in part also be explained by genetic influences (Couture et al. 2014; Holeski et al. 2012; Moore et al. 2014) or the important finding that PSM strategies vary with both plant stage and age (Boege and Marquis 2005). This is further complicated by the fact that the longevity of a stage can vary extensively for a given taxa, due to variation in external pressures (Barthélémy and Caraglio 2007). Barton and Koricheva (2010) reviewed the literature, and found that PSM concentrations generally increase with age for the seedling, but not the juvenile stage of woody plants. Most studies on PSM in woody plants seem to be conducted on seedlings, probably for practical reasons, and data are limited for later plant stages. A first step towards obtaining the knowledge needed is to gather empirical evidence so that we can start to elucidate prevalent patterns.

In this study, we examine how PSM concentrations in foliage from an abundant woody species in the northern boreal forest of Eurasia, the downy birch (*Betula pubescens* Ehrh.), vary with plant age. We made use of a natural experiment facilitated by a commonplace temporal disturbance factor, i.e. forest clear-cutting, by sampling trees considered undamaged and growing on 5, 10 and 15 years old clear-cuts. We studied birch because the genus dominates regrowth on boreal forest clearings, and its ontogeny is considered typical for pioneer species (Kimmins 2003). In PSM studies, a wish to understand all PSM patterns in the system must be traded against the need for interpretable results. We opted to focus on phenolics, specifically low molecular weight phenolics and condensed tannins. These PSMs are known to vary in boreal foliage due to factors strongly affected by clear-cutting such as soil nutrients (Hakulinen et al. 1995), UV radiation (Julkunen-Tiitto et al. 2015), and herbivory (Stolter 2008), but the direct temporal influence of this prevalent activity remains largely unstudied.

## Materials and Methods

### Study Area

The study areas are located 100 km apart within the boreal forest zone of the Oslofjord region in Southern Norway (SandeW at 59°42′N, 10°7′E hereafter termed west, and Rakkestad at 59°30′N, 11°22′E hereafter termed east). Both areas are lowland boreal forest belonging to the same climatic zone (Moen 1999), which should minimise environmental variance other than that of our focal interest (plant age). The climate is continental with cold winters (February norm −4.5 °C in west and −5.6 °C in east) and warm summers (June norm 14.9 °C in west and 13.7 °C in east) (Norwegian Meteorological Institute 2013). Start of growing season (first day of the year with mean temperature > 5 °C) is 2 May (west) and 25 April (east). Normal precipitation during June is 59 mm (west) and 65 mm (east). Norms are based on the years 1961–1990. The quaternary geology is mainly marine sediments on gneiss and granite rock. All sampling sites had intermediate soil fertility, i.e. a site index of G14-G17 (see Tveite 1977 for details on the H_40_ index, which indicates tree height when trees are 40 years at breast height = 1.3 m). A clear-cut on these sites typically produces about twice as much birch biomass per unit area as clear-cuts on less fertile sites (Wam et al. 2010).

The forest is commercially cultivated with mainly Norway spruce *(Picea abies)*, which, to a large extent, is also the naturally dominant conifer tree species in the region. There are smaller areas of Scots pine (*Pinus sylvestris*), primarily on drier sites of poor site quality, while deciduous trees are intermixed throughout. Forest clearings have a strong upsurge of mainly birch (*Betula spp*), which makes up 78% of the browse biomass for moose on typical clear-cuts (<20 years old, intermediate site quality) in west, compared to 95% in east (Wam et al. 2010). Grasses dominate the field layer of these clearings, while bilberry (*Vaccinum myrtillus*) is the most abundant field layer plant in the older forest (detailed vegetation data given in Wam and Hjeljord 2010). Forest clearing on the sampled study sites had been carried out by clear-cutting (stems only, no whole-tree harvesting) at the commercially mature stage using harvesters and forwarders. There was no use of herbicides, scarification, fertilizing or other silviculture on the study clear-cuts. Clear-cuts in the region are small in global comparison (median 1.6 ha, range 0.2–14.4). Because the sample sites stemmed from clear-cutting mature forest stands, we assume that the age of birch trees on the clear-cut corresponds to the age of the clear-cut.

### Foliage Sampling

We sampled foliage between 24 June 2013 and 12 July 2013, alternating sampling between age classes as well as the two study areas to avoid date bias. The June temperature in 2013 was very close to the 1960–1990 norms, while the precipitation was somewhat higher than the norms: 13.8 °C and 142 mm in west, and 13.6 °C and 134 mm in east (Norwegian Meteorological Institute 2013). We used an approach akin to stratified random sampling: Sample clear-cuts (*N* = 24 per area) were randomly drawn from all available G14-G17 spruce forest clear-cuts with an age of 5, 10 or 15 years since logging (i.e. age class as strata, with eight random replicates per strata). The trees to be sampled for foliage were systematically selected by pacing out given distances along a fixed cross-section of the clear-cut, and sampling the nearest undamaged trees when the set distance was reached (i.e. random selection of individuals), leaving a buffer zone <10 m from the clear-cut edge. To further avoid observer bias when sampling trees, we ensured our path was not diverted by any obstacles, like ditches or piles of logging waste. The average heights of our sample trees were 75 ± 6.9, 108 ± 7.9 and 154 ± 10.2 cm (mean ± 1SE) per age class (5, 10, 15 years). On each clear-cut, we defoliated the apex shoot along the outer 20–30 cm of the current year’s growth (mimicking browsing by moose, the main co-adapted large herbivore in the area) on nine trees considered to be undamaged (no obvious signs of herbivory, disease or mechanical damage)*.* Composite samples were combined of the foliage from all the trees sampled within a clear-cut and sealed in plastic bags on site. Upon return to the field quarters in the afternoon, we left the samples to air-dry in open paper traces inside a cabin with no exposure to sunlight. After 3–5 days, the foliage had a dry matter concentration of 91.2 ± 0.15%. Note that we also opportunistically sampled 3 ± 0.2 trees/clear-cut with signs of herbivory from the current summer, but no previous damage. These were also combined into composite samples, sealed on site and kept separate from the undamaged samples. In the data analyses, we include these samples because they followed the same age pattern as the undamaged trees (i.e. there was no significant herbivory: age interaction effect), and they strengthen our sample size.

### Chemical Analyses

We measured concentrations of low molecular weight phenolics on the air-dried foliage samples (compounds listed in the Electronic Supplementary Material, Table S1). We took three subsamples from each sample, and used the mean of subsamples to represent the original sample. We found an outlying value for MeOH-insoluble condensed tannins in one such set of subsamples, and excluded that particular measurement (i.e. mean calculated from two, not three subsamples). The plant material was ground into fine powder using a Retsch MM400 ball mill (Retsch, Haag, Germany). From the resulting powder, we determined total carbon (C) and nitrogen (N) with a Micro Cube (Elementar Analysen, Hanau, Germany), using 5–6 mg plant material. For the phenolic analysis, further subsamples of *c.*10 mg were extracted with 600 μl methanol (MeOH) and homogenised for 20 s on a Precellys 24 homogeniser (Bertin Technologies, Montigny-le-Bretonneux, France). We thereafter cooled samples on ice for 15 min before centrifuging, and transferred the supernatant to a 10 ml glass tube. The residue was again extracted with 600 μl MeOH, homogenised, centrifuged and the supernatant removed, and the same extraction process was conducted twice more until both the residue and the supernatant were completely colourless. We evaluated the extraction procedure by initially testing five samples for any residual HPLC phenolics and condensed tannins. The combined supernatants were evaporated in a vacuum centrifuge (Eppendorf concentrator plus; Eppendorf, Hamburg, Germany), sealed, and stored in a freezer (−20 °C) until the high performance liquid chromatography (HPLC) analysis. The residues were also stored in a freezer for further analysis of the insoluble condensed tannins.

The dried extracts were dissolved in 200 μl MeOH and diluted with 200 μl ultra-clean water. Low molecular weight phenolics were analysed using a HPLC system (Agilent Series 1200, Agilent Technologies, Waldbronn, Germany) with a G1312A binary pump, a G1329A autosampler, a G1316A thermoregulated column heater, and a G1315D diode array detector. For the stationary phase, we used a Thermo Scientific column type (Thermo Fisher Scientific Inc., Waltham, USA), with a 50 × 4.6 mm internal diameter and filled with ODS Hypersil (3 μm) particles. The mobile phase consisted of two solvents that eluted the samples by means of a gradient as in Nybakken et al. (2012). The injection volume was 20 μl. The absorbance spectra at 270 and 320 nm, along with respective retention times, were used to identify the chemical compounds and to calculate concentrations by comparison with the commercial standards listed in the Electronic Supplementary Material, Table S2.

We analysed condensed tannins from the HPLC extract (soluble fraction, referred to as ‘MeOH-soluble condensed tannins’ throughout the text) and from the dried residue after phenolic extractions (insoluble, cell wall bound fraction, referred to as ‘MeOH-insoluble condensed tannins’) with the acid butanol assay (Hagerman 2002). We calculated these concentrations using purified condensed tannins (according to Hagerman 2002) from *Betula nana* (dwarf birch) leaves. Analyses of the insoluble fraction for 15 years old clear-cuts in the east was not conducted. Mean ± 1SE for all compounds are given per area and age in Table S1.

### Data Analyses

We analysed the variance of PSM concentrations using generalised linear models (‘glm’ in R, version 3.2.0, The R Foundation for Julkunen Computing), with ‘age of clear-cut’ (5, 10, 15 years) and ‘area’ (east, west) as predictors. The homogeneity of response variances across each predictor was checked with residual plots from exploratory linear fits (Zuur et al. 2007), and found adequate. As stated under ‘foliage sampling’, we also had 3 ± 0.2 trees/clear-cut with signs of recent moose browsing (otherwise undamaged). We included these samples in the data analyses, and tested for the interaction effect of ‘age of clear-cut’ and ‘herbivory’ (yes, no). These were clearly non-significant, and temporal patterns were visually identical to samples considered to be undamaged. We therefore opted to merge the two sets of samples to increase our sample size. The statistical analyses thus were ran at the level of clearcuts, i.e. a sampling unit containing all foliage from each of 24 clearcuts within the two study areas (*N* = 48 samples in total).

Each response parameter (concentration of carbon, nitrogen or a given PSM group) was tested in separate models with predictors and their interaction as fixed effects. We first used ordinary linear fits and thereafter logit link function and binomial distribution for proportional data (McCullagh and Nelder 1989) (quasi-correction for overdispersion, Zuur et al. 2009). Because the two sets of models gave the same results, we present data from the linear fits, as its model coefficients can be straightforwardly interpreted without back-transformation. We ranked candidate models (i.e. the full model with all predictors and partial models with fewer predictors) by ΔAICc and AICc weights (Burnham and Anderson 2002). We validated all candidate and final models by the lack of patterns in plots of residuals against fitted values and QQ plots of standardized residuals (Zuur et al. 2007).

In order to examine co-variation between PSM compounds, and to better visualise compositional changes in their concentrations with age, we ran principal component analyses (PCA) (‘prcomp’ in R). The differences in concentrations were large between some compounds (low molecular weight phenolics versus tannins), so we centred and scaled observations prior to the PCA (van den Berg et al. 2006).

## Results

**Fig. 1:**
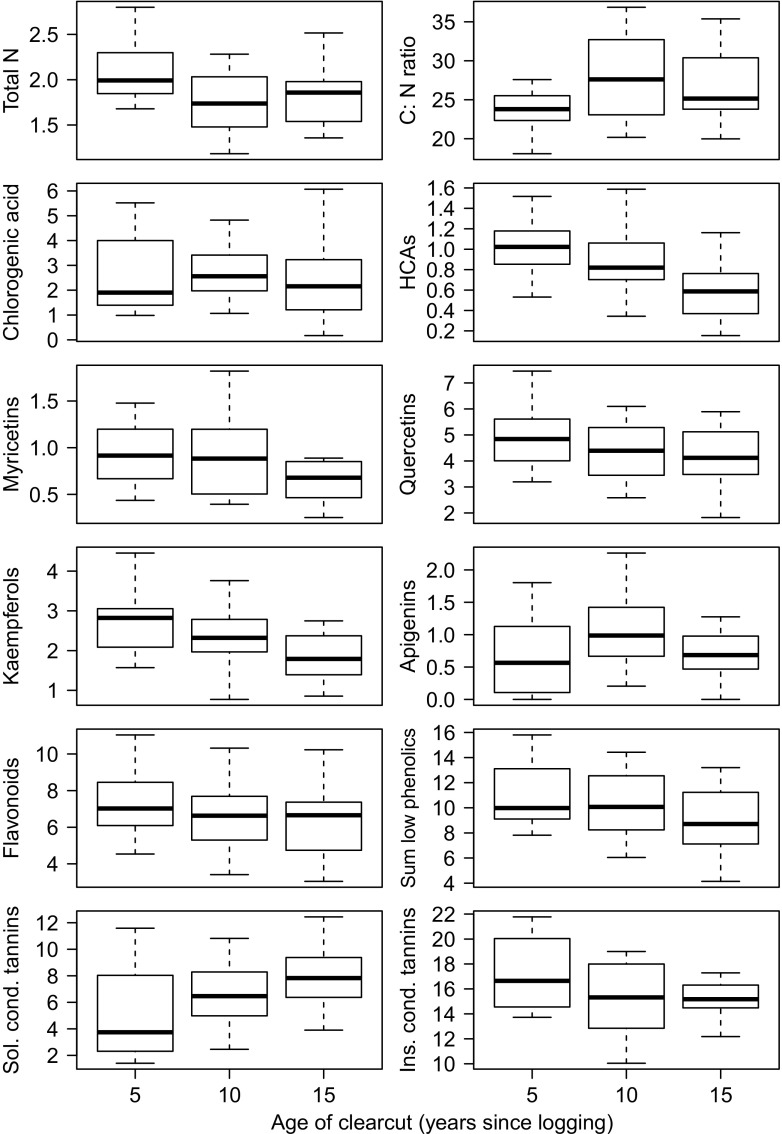
Concentrations of carbon (%), nitrogen (%) and phenolics (mg g^−1^ DW) in birch foliage growing on clear-cuts of varying age: 5, 10 and 15 years since logging (mean 1 SE tree height 75 ± 6.9 cm, 108 ± 7.9 cm and 154 ± 10.2 cm) in two boreal forests of intermediate site fertility, southern Norway in summer 2013 (composite samples of foliage from 12 ± 0.2 trees per 48 clear-cuts, dry matter 91.2 ± 0.15%). Note that for MeOH-insoluble tannins, data from 15 years old clear-cuts is from one area only (west). Abbreviations: “Sum low phenolics” = all low molecular weight phenolics measured (see Table S1), “Sol. cond. tannins” = MeOH-soluble condensed tannins, “Ins. cond. tannins” = MeOH-soluble condensed tannins

Concentrations of total nitrogen, but not total carbon, were higher in foliage from 5 years old clear-cuts compared to 10 and 15 years old clear-cuts (Table 1). Subsequently, the C: N ratio increased with clear-cut age (Fig. 1). The concentrations of several phenolic groups showed a downward trend with age. This applied most strongly to phenolic acids (apart from chlorogenic acid), and, to a lesser extent, the three flavonoid glycoside groups (combined concentrations of different glycosides) kaempferols, myricetins and quercetins. The concentrations of one apigenin glycoside (see the Electronic Supplementary Material, Table S1) and chlorogenic acid did not significantly change with age. In sum, the low molecular weight phenolics decreased with age. The two fractions of condensed tannins showed a different pattern: the MeOH-soluble fraction doubled from 5 to 15 years, while the MeOH-insoluble fraction showed no significant change. There were also area differences in the C: N ratio (being lower in the west than in the east) as well as most PSM compounds (generally higher in the west) (Table 1). Most notably, MeOH-soluble condensed tannins were twice as high in the west, and MeOH-insoluble condensed tannins were twice as high in the east.

**Table 1:** Concentrations of carbon (%), nitrogen (%) and phenolics (mg g^−1^ DW) in birch foliage growing on clear-cuts of varying age: 5, 10 and 15 years since logging (mean ± 1 SE tree height 75 ± 6.9 cm, 108 ± 7.9 cm and 154 ± 10.2 cm) in two boreal forests (west, east) of intermediate site fertility, southern Norway in summer 2013 (composite samples of foliage from 12 ± 0.2 trees per 48 clear-cuts^a^). Tests were run as sequential contrasting against the reference level east, 5-year old (= intercept *α*). Single coefficients must be interpreted in relation to this reference level and eventual interaction effects. Only significant (95% level) or near-significant coefficient values are shown

	Coefficients [*t*, *p-value*]^*b*^			
Response	*α*	*β*_*1*_ *(age)*	*β*_*2*_ *(area)*	*β*_*1*_ * β_*2*_
Total nitrogen	1.9	−0.03 [−3.1, 0.003]	0.4 [4.4, 0.000]	n.s
Total carbon	46.2	n.s	n.s	n.s
C: N ratio	24.3	0.4 [2.8, 0.007]	−4.7 [−4.2, 0.000]	n.s
Myricetins	1.1	−0.03 [−2.0, 0.053]	0.2 [1.7, 0.089]	n.s
Quercetins	4.8	−0.1 [−1.6, 0.109]	1.1 [3.0, 0.004]	n.s
Apigenins	1.0	n.s	−0.3 [−2.2, 0.035]	n.s
Kaempferols	3.6	−0.1 [−4.2, 0.000]	−0.7 [−1.4, 0.156]	0.1 [2.0, 0.053]
∑ flavonoids^c^	7.6	−0.1 [−1.7, 0.090]	1.0 [1.7, 0.103]	n.s
Hydroxycinnamic acids (HCAs)	1.2	−0.04 [−3.9, 0.000]	n.s	n.s
Chlorogenic acid	1.6	n.s	2.0 [7.1, 0.000]	n.s
∑ low molecular weight phenolics^d^	11.2	−0.2 [−2.2, 0.035]	3.1 [3.6, 0.001]	n.s
MeOH-soluble condensed tannins	2.5	0.2 [2.8, 0.007]	3.7 [0.7, 0.000]	n.s
MeOH-insoluble condensed tannins^e^	30.6	n.s	−12.0 [−3.1, 0.004]	n.s

^a^Clear-cuts were randomly chosen from all available clear-cuts with age 5, 10 or 15 years since clearing (8 replicates of each age/area)

^b^Ordinary linear model (no transformations applied). Generalized models with logit link and binomial correction (quasi-binomial, approximated Wald-statistics) gave consistently the same results

^c^∑ flavonoids = myricetins + quercetins + apigenins + kaempferols

^d^∑ low molecular weight phenolics = ∑ flavonoids + chlorogenic acid + HCA

^e^Note that for MeOH-insoluble tannins, there are no samples for 15 years old clearcuts in the eastern area

**Fig. 2:**
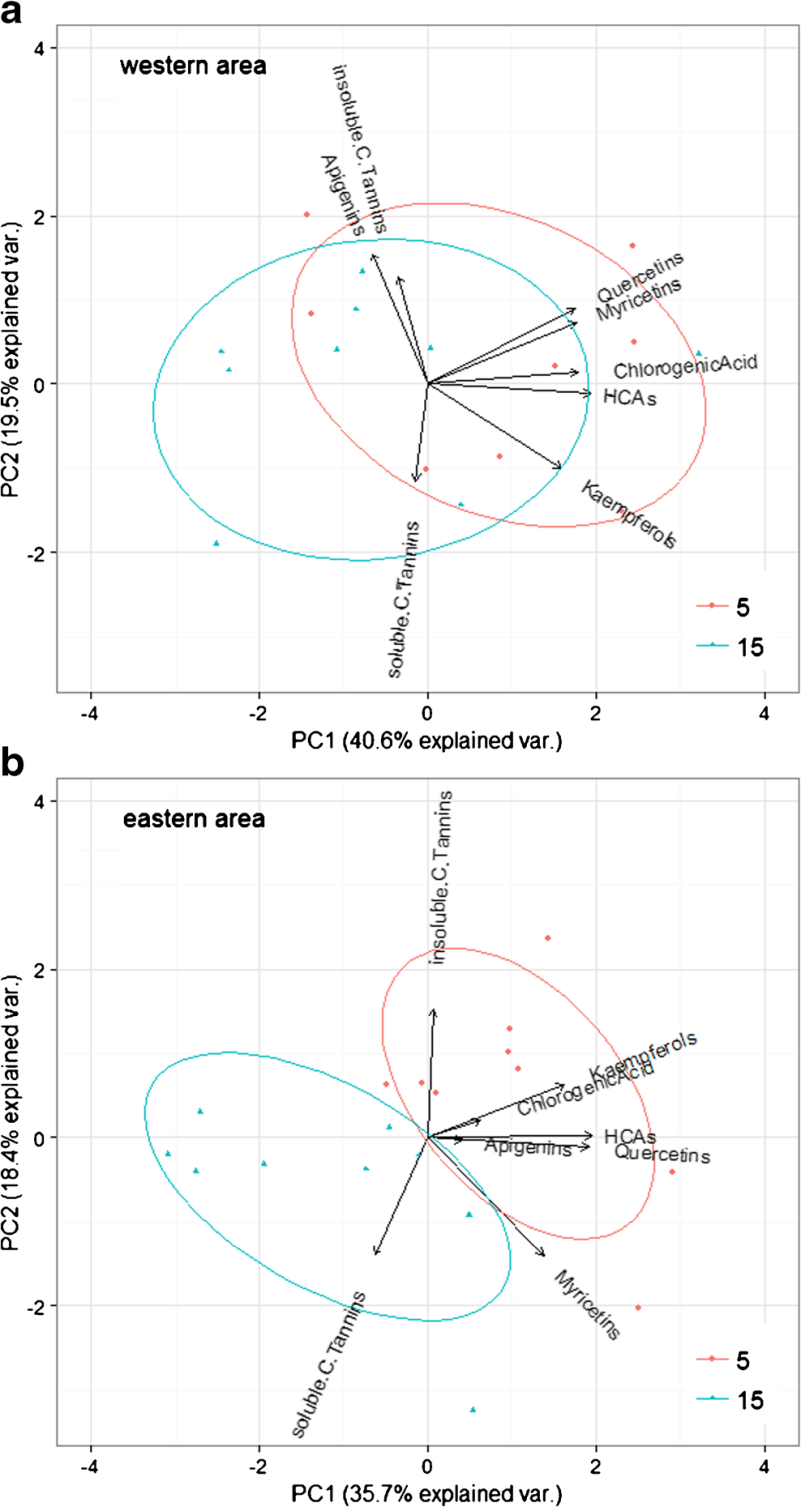
Co-variance among phenolic concentrations in birch foliage on clear-cuts 5 and 15 years of age (mean ± 1 SE tree height 75 ± 6.9 cm and 154 ± 10.2 cm) in two boreal forests of intermediate site fertility, southern Norway in summer 2013 (composite samples of foliage from 12 ± 0.2 trees per 48 clear-cuts). Note that for MeOH-insoluble tannins, data from 15 years old clear-cuts is from one area only (west). Compounds on arrows close together co-vary the most, and in a differing direction than other such clusters. Ellipses (the “circles” around observations) are 2/3 confidence intervals. There was a shift from an array of co-varying low molecular weight phenolics to a concentration of tannins with age, most notably MeOH-soluble condensed tannins

The shift in PSM composition from low molecular weight phenolics at younger age to tannins at older age were clearly visible in PCA bi-plots, and more pronounced for the eastern area (Fig. 2). Three main axes appeared: 1) low molecular weight phenolics 2) the MeOH-insoluble condensed tannins and 3) the MeOH-soluble condensed tannins (their corresponding principal components are given in the Electronic Supplementary Material, Table S3). Practically all low molecular weight phenolics co-varied strongly at all ages. They clustered in the opposite direction of the condensed tannins along PCA1 (which captured 35–40% of the variance). PCA2 mainly split the two fractions of condensed tannins (explaining an additional 18–20% of the variance).

## Discussion

In this study, the sum and most group concentrations of low molecular weight phenolics in birch foliage decreased with plant age (as sampled from 5, 10 and 15 years old clear-cuts in boreal forests). These groups and compounds also co-varied similarly at all ages, suggesting a lack of temporal compound-specific prioritisation of such phenolics. In contrast, concentrations of MeOH-soluble condensed tannins increased with plant age. The compositional shift possibly lends support to several established hypotheses that may provide proximate explanations for these temporal patterns. We will first discuss our findings in relation to resource constraints (“bottom-up” hypotheses), and then external pressures (“top-down” hypotheses).

The element most limiting for boreal forest plants is nitrogen (Vitousek and Howarth 1991), while carbon, the main constituent of living matter, is readily available from CO_2_ (Livingston and Beall 1934). The deciduous upsurge after forest clearing (an assart effect, Kimmins 2003; Odum 1969) coincides with a rapid, short-term elevation of soil nitrate (Kreutzweiser et al. 2008; Mattson 1980). When nitrogen concentration drops back down (suggested to occur already by three to five years in boreal forests, Prescott 2002), carbon surplus in the forest plants increases. According to, for example, the ‘protein competition’ model by Jones and Hartley (1999), phenolic synthesis should take priority over growth under N-limited conditions. Such relationships are extensively supported by fertilizer experiments with woody species of boreal plants, but the effect on PSM allocation is strongly dosage-dependent (Hakulinen et al. 1995) and may vary with multiple factors such as plant species (Stolter et al. 2010), light (Hemming and Lindroth 1999), genotype (Gebauer et al. 1998), the sex of the plant (Randriamanana et al. 2014), and ontogeny (Orians et al. 2010).

In our study, the decreasing total N concentration with age observed in the birch foliage fits well with the preceding arguments. Because the carbon concentrations did not decrease, the C: N ratio increased with age, indicating that the growth rate had decreased relative to carbon storage (Ågren 2004). Changes in plant water balance may also have contributed to this. In addition to increased competition among plant individuals, increased crown coverage affects water filtration (Lambers et al. 1998). The latter may further influence assart effects by altering the wet deposition of atmospheric N (Prescott 2002). While practically all low molecular weight phenolics identified in our foliage decreased with age, this was countered by an increase in MeOH-soluble condensed tannins. Consequently, the total concentration of all phenolics we measured did not change (in the eastern area it was bell-shaped) (Table S1). Our study thus provides support for PSM allocations being driven by resource constraints, specifically nitrogen.

Donaldson et al. (2006), one of the few studies of long-term age patterns of foliage PSM allocations (albeit in a different species, *Populus tremuloides*) found the same compositional changes as in our study: low molecular weight phenolics (glycosides) decreased with plant age, while condensed tannins increased over the first five years before remaining fairly constant among older age classes. Condensed tannins are suggested to be final stages of biosynthetic phenolic pathways (Winkel-Shirley 2002), and their increase necessarily leads to a reduction of lower stage phenolic compounds, unless there is a sufficient input of new resources for allocation to the latter. Couture et al. 2014 show that environmental factors may affect the phenolic versus tannins trade-off across ontogenetic stages of perennial plants (*Populus tremuloides*): elevated CO_2_ levels produced more tannins and less phenolics with age, while elevated O_3_ produced the opposite pattern.

Needless to say, the biosynthetic trade-offs a plant makes between low and high molecular weight compounds can be strongly situational. Nurmi et al. (1996), for example, found that the between-tree variation in phenolic composition in birch was higher than the seasonal variation. It has also been frequently documented that tannin concentrations and structures may vary not only temporally with foliage development (Macauley and Fox 1980; Riipi et al. 2002; Schultz et al. 1982), but also spatially within plants individuals (Schultz et al. 1982), within plant populations (Hunter et al. 1996; Laitinen et al. 2000) and across years (Covelo and Gallardo 2001; Laitinen et al. 2000). Our study adds to this as we also found that several phenolic compounds in a specific plant species varied between areas, despite our sampling being designed to minimise confounding effects of site (e.g. site fertility, climate, weather).

On young clear-cuts with little to no crown coverage, light is an external stress factor rather than a resource constraint for the growing plants (Lambers et al. 1998). Because increasing crown coverage increases light filtration to lower branches, the effects of light stress are likely to decrease with plant age (e.g. Tucker et al. 1987). Phenolic compounds, especially those located in epidermal cells, serve as sunscreens against ultraviolet and blue light (Lois 1994), and/or as antioxidants (Bi and Felton 1995). In *Betula* spp., it is well established that UV-B radiation induces quercetins, with more scant evidence of responses in other low molecular weight phenolics as well as in condensed tannins (reviewed by Julkunen-Tiitto et al. 2015). The decrease of low molecular weight phenolics observed with clear-cut age in our study may thus be a response to reduced light stress.

The compositional changes from low molecular weight phenolics to condensed tannins in our study may also be related to herbivory risk. In our study system, birch is staple food for a co-adapted and abundant large herbivore (moose *Alces alces*, Wam and Hjeljord 2010). The risk of browsing is higher when the plant height reaches the chest height of the herbivore (Bobrowski et al. 2015), which for moose occurs near the oldest clear-cut class (see tree heights in methods). For moose, condensed tannins reduce the digestibility of protein in *Betula* spp. (Spalinger et al. 2010). As a counter-strategy, though, moose produce salivary tannin-binding proteins (Hagerman and Robbins 1993; Juntheikki 1996). It is not known whether tannins actually deter moose from browsing (e.g. Hjeljord et al. 1990). Hares (*Lepus timidus*) are another mammal herbivore in the area. *Betula* spp. may comprise a substantial part of the summer diet of hares in Canada (Seccombe-Hett and Turkington 2008), but this has not been investigated in Scandinavia. In winter, low molecular weight phenolics in birch twigs are considered a greater deterrent to hares than condensed tannins (Bryant et al. 1992). If this applies to the foliage as well, it may partly explain our finding that the concentration of these PSMs decreased with age (and the plants grew out of reach of hares). Additionally, birch is susceptible to insect herbivory at all ages, which makes for complex herbivory interactions (Danell and Huss-Danell 1985; Olofsson et al. 2007). Clearly, there are ample herbivory pressures that may in part explain ontogenetic strategies of PSM defences in our study plants. We need far more targeted studies to verify and determine their different roles.

## Conclusions

Our study shows a compositional shift from low molecular weight phenolics to MeOH-soluble condensed tannins in birch foliage with plant age (as sampled from 5, 10 and 15 years old clear-cuts in boreal forests). The results fit well with several hypotheses that may provide proximate explanations for age patterns in PSM allocations, including both resource constraints and external pressures. Regardless of these explanations, our study adds an important perennial perspective (plant age) to temporal PSM patterns already well-known in studies of boreal plant phenology (foliage age).

## Electronic supplementary material

ESM 1 (PDF 673 kb)
