# Supplementary material for: Compositional Changes in Foliage Phenolics with Plant Age, a Natural Experiment in Boreal Forests
Source: J Chem Ecol. 2017 Aug 29;43(9):920–8. doi: 10.1007/s10886-017-0881-5 (PMC5636854; doi:10.1007/s10886-017-0881-5)
Supplement: Supplementary file 1 — (PDF 673 kb) [file 10886_2017_881_MOESM1_ESM.pdf]

Wam HK, Stolter C, Nybakken L (2017). Compositional changes in foliage phenolics with plant age, a natural experiment in boreal forests. *Journal of Chemical Ecology, submitted manuscript*. Supplementary material.

Table S1. Phenolic concentrations (mg g^{-1} DW) (mean ± 1 SE) in foliage from undamaged[a] birch *Betula pubescens* on clearcuts (*N* = 48[b]) of varying age in two boreal forests of intermediate site fertility, Norway summer 2013.

	**Eastern area**			**Western area**		
Clear-cut age	**5**	**10**	**15**	**5**	**10**	**15**
Phenolic acids						
Chlorogenic acid	1.50±0.13	2.02±0.22	1.37±0.30	4.63±0.80	3.75±0.45	3.28±0.40
Hydroxycinnamic acid der 1	0.06±0.01	0.09±.02	0.05±0.01	0.44±0.07	0.37±0.06	0.27±0.04
Hydroxycinnamic acid der 2	0.56±0.05	0.48±0.05	0.27±0.06	0.44±0.07	0.37±0.06	0.27±0.04
Hydroxycinnamic acid der 3	0.09±0.01	0.07±0.01	0.05±0.01	0.09±0.01	0.08±0.01	0.06±0.01
Hydroxycinnamic acid der 4	0.06±0.003	0.07±0.01	0.04±0.01	0.04±0.01	0.05±0.01	0.04±0.01
Hydroxycinnamic acid der 5	0.04±0.003	0.06±0.01	0.03±0.01	0.05±0.01	0.06±0.01	0.05±0.01
Hydroxycinnamic acid der 6	0.02±0.003	0.04±0.01	0.01±0.003	0.02±0.004	0.02±0.01	0.02±0.004
Hydroxycinnamic acid der 7	0.02±0.01	0.03±0.01	0.02±0.004	0.02±0.01	0.03±0.01	0.01±0.003
Hydroxycinnamic acid der 8	0.02±0.002	0.05±0.02	0.01±0.005	0.01±0.002	0.02±0.004	0.02±0.01
Hydroxycinnamic acid der 9	0.04±0.02	0.03±0.01	0.02±0.01	0.02±0.01	0.01±0.01	0.01±0.004
Hydroxycinnamic acid der 10	0.04±0.01	0.06±0.01	0.03±0.01	0.03±0.003	0.04±0.01	0.03±0.004
Hydroxycinnamic acid der 11	0.02±0.004	0.03±0.01	0.02±0.01	0.05±0.01	0.03±0.01	0.02±0.004
Sum phenolic acids	**2.57±0.16**	**2.99±0.32**	**1.90±0.32**	**5.52±0.81**	**4.57±0.57**	**3.94±0.47**
Flavonoids						
myricetins						
myricetin3galactoside+glucoronide	0.68±0.12	0.64±0.11	0.57±0.15	1.20±0.27	0.90±0.11	0.69±0.09
myricetin glycoside 1	0.21±0.04	0.14±0.03	0.11±0.04	0.30±0.15	0.16±0.04	0.09±0.01
Sum myricetins	0.89±0.15	0.78±0.14	0.68±0.18	1.50±0.41	1.06±0.13	0.77±0.10
quercetins						
quercetin3galactoside	1.30±0.22	0.75±0.14	0.97±0.31	1.39±0.18	1.04±0.17	0.91±0.08
quercetin3glucoside	1.97±0.12	2.30±0.17	1.66±0.28	2.70±0.23	2.94±0.39	2.95±0.33
quercetin3glucuronide	0.36±0.07	0.19±0.04	0.23±0.08	0.37±0.08	0.34±0.11	0.21±0.05
querc3arabinofuranoside	0.42±0.04	0.46±0.04	0.48±0.10	0.77±0.07	0.78±0.15	0.71±0.03
quercetin glycoside 1	0.22±0.08	0.13±0.03	0.07±0.03	0.51±0.24	0.14±0.04	0.55±0.05
quercetin glycoside 2	0.36±0.03	0.30±0.04	0.17±0.03	0.11±0.02	0.14±0.04	0.14±0.02
Sum quercetins	4.54±0.26	4.09±0.35	3.54±0.42	5.40±0.50	5.11±0.72	4.95±0.42
kaempferols						
kaempferol3glucoside	0.43±0.05	0.37±0.03	0.36±0.05	0.58±0.10	0.40±0.06	0.37±0.05
kaempferol3glucuronide	0.46±0.04	0.50±0.03	0.34±0.07	0.83±0.11	0.73±0.10	0.68±0.06
kaempferl3rhamnoside	0.20±0.05	0.32±0.08	0.38±0.07	0.52±0.14	0.65±0.16	0.36±0.08
kaempferol glycoside 1	0.87±0.17	0.67±0.08	0.39±0.07	0.76±0.14	0.83±0.09	0.57±0.08
kaempferol glycoside 2	0.57±0.11	1.02±0.21	-	0.52±0.13	0.68±0.21	0.57±0.06
kaempferol glycoside 3	0.68±0.21	-	-	0.32±0.25	-	0.25±0.08
Sum kaempferols	2.86±0.30	2.26±0.18	1.50±0.20	2.59±0.21	2.79±0.47	2.17±0.14
apigenins						
apigenin glycoside 1	0.14±0.03	0.15±0.02	0.09±0.01	0.28±0.05	0.21±0.03	0.15±0.02
apigenin glycoside 2	0.91±0.35	1.23±0.13	0.67±0.09	0.47±0.15	0.84±0.32	0.62±0.13
Sum apigenins	0.86±0.21	1.38±0.14	0.77±0.09	0.53±0.14	0.91±0.32	0.67±0.12
naringenin	0.39±0.06	0.30±0.04	0.28±0.04	0.13±0.04	0.20±0.10	0.21±0.04
eriodictyol	0.20±0.04	0.16±0.02	0.16±0.03	0.35±0.12	0.17±0.04	0.32±0.06
luteolin glycoside	0.05±0.01	0.05±0.01	0.05±0.01	0.06±0.02	0.08±0.03	0.07±0.02
Sum, flavonoids	**6.93±0.37**	**6.76±0.62**	**5.48±0.64**	**7.84±0.76**	**7.41±1.23**	**6.82±0.58**
Sum, low molecular weight phenolics	**9.50±0.39**	**9.75±0.88**	**7.37±0.90**	**13.36±1.37**	**11.98±1.73**	**10.76±1.00**
MeOH-soluble condensed tannins	**2.49±0.28**	**5.88±0.93**	**6.37±1.24**	**8.33±1.19**	**8.71±1.53**	**9.04±0.52**
MeOH-insoluble condensed tannins	**30.52±1.20**	**34.84±2.66**	**29.13±0.89**	**17.24±2.61**	**16.96±2.67**	**15.18±0.50**

[a] Foliage was analysed per clearcut as a composite sample of 9 randomly chosen trees with no sign of herbivory, disease or mechanical damage.
[b] Randomly drawn from all available clearcuts of age 5, 10 or 15 years since clearing (8 replicas for each age class).

Table S2. List of commercial standards used to identify and compute concentrations of phenolic compounds. All standards are supplied by Sigma-Aldrich (St. Louis, USA)

**Standards**	**Applied to the following compounds**
Chlorogenic acid	Chlorogenic acid; *p*-OH-cinnamic acids
Kaempferol3glucoside	Kaempferol glycosides
Myrectin3rhamnoside	Myricetin glycosides
Quercetin3glucoronide	Quercetin glycosides
Apigenin7glucoside	Apigenin glycosides
Luteolin7glucoside	Luteolin glycoside
Naringenin	Naringenin
Eriodictyol	Eriodictyol

Table S3. Principal components of phenolic concentrations (mg g^{-1} DW) (mean ± 1 SE) in foliage from undamaged[a] birch *Betula pubescens* on clearcuts (*N* = 48[b]) of varying age in two boreal forests of intermediate site fertility, Norway summer 2013. PCAs are shown for each age class separately (areas combined), and for each area separately (5 and 15 years combined, which is the dataset underlying Fig. 2 in the main manuscript).

	**Eastern area + Western area**					
Clear-cut age 5 years	**PCA1**	**PCA2**	**PCA3**	**PCA4**	**PCA5**	**PCA6**
Myricetins	0.253	0.393	-0.489	-0.269	0.559	0.123
Quercetins	0.341	0.390	-0.234	0.237	-0.513	0.528
Apigenin glycosides	-0.306	-0.066	-0.588	0.675	0.065	-0.295
Kaempferols	0.042	0.419	0.573	0.549	0.432	0.081
Hydroxycinnamic acids (HCAs)	-0.047	0.628	0.034	-0.220	-0.251	-0.670
Chlorogenic acid	0.490	-0.146	0.121	0.233	-0.309	-0.292
MeOH-soluble condensed tannins	0.457	-0.309	0.019	-0.020	0.249	-0.194
MeOH-insoluble condensed tannins	-0.523	0.053	0.127	-0.106	-0.117	0.200
Proportion of variance	**0.395**	**0.249**	**0.136**	**0.079**	**0.064**	**0.038**

	**Eastern area + Western area**					
Clear-cut age 10 years	**PCA1**	**PCA2**	**PCA3**	**PCA4**	**PCA5**	**PCA6**
Myricetins	0.417	-0.191	-0.324	-0.125	-0.675	0.354
Quercetins	0.468	-0.006	-0.144	0.345	-0.187	-0.343
Apigenin glycosides	0.351	0.449	-0.094	0.086	0.057	-0.583
Kaempferols	0.259	0.049	0.871	-0.265	-0.256	-0.051
Hydroxycinnamic acids (HCAs)	0.431	0.265	0.022	-0.262	0.319	0.345
Chlorogenic acid	0.397	-0.249	0.159	0.545	0.438	0.354
MeOH-soluble condensed tannins	0.270	-0.451	-0.210	-0.622	0.375	-0.254
MeOH-insoluble condensed tannins	0.030	0.651	-0.192	-0.189	0.078	0.325
Proportion of variance	**0.489**	**0.261**	**0.112**	**0.062**	**0.031**	**0.023**

	**Eastern area + Western area**					
Clear-cut age 15 years	**PCA1**	**PCA2**	**PCA3**	**PCA4**	**PCA5**	**PCA6**
Myricetins	0.335	-0.464	-0.406	0.007	-0.068	0.130
Quercetins	0.422	-0.134	0.406	-0.341	0.000	0.054
Apigenin glycosides	0.008	-0.566	0.568	0.494	-0.218	-0.169
Kaempferols	0.373	0.299	0.186	0.276	0.664	-0.381
Hydroxycinnamic acids (HCAs)	0.374	-0.347	-0.243	-0.405	0.080	-0.418
Chlorogenic acid	0.435	0.171	0.288	-0.118	-0.019	0.659
MeOH-soluble condensed tannins	0.368	0.034	-0.413	0.620	-0.040	0.203
MeOH-insoluble condensed tannins	-0.325	-0.455	-0.027	-0.028	0.706	0.395
Proportion of variance	**0.502**	**0.206**	**0.104**	**0.090**	**0.049**	**0.027**

	**Eastern area**			**Western area**		
Clear-cut age 5+15 years	**PCA1**	**PCA2**	**PCA3**	**PCA1**	**PCA2**	**PCA3**
Myricetins	0.382	-0.545	-0.104	0.438	0.264	-0.283
Quercetins	0.532	-0.044	0.017	0.433	0.323	0.032
Apigenin glycosides	0.116	-0.011	0.040	-0.162	0.555	-0.258
Kaempferols	0.450	0.244	0.091	0.395	-0.362	-0.212
Hydroxycinnamic acids (HCAs)	0.543	0.009	-0.106	0.483	-0.042	0.223
Chlorogenic acid	0.174	0.079	0.770	0.443	0.050	-0.039
MeOH-soluble condensed tannins	-0.175	-0.539	0.549	-0.036	-0.416	-0.775
MeOH-insoluble condensed tannins	0.017	0.587	0.271	-0.086	0.457	-0.394
Proportion of variance	**0.357**	**0.184**	**0.171**	**0.406**	**0.195**	**0.130**

[a] Foliage was analysed per clearcut as a composite sample of 9 randomly chosen trees with no sign of herbivory, disease or mechanical damage.
[b] Randomly drawn from all available clearcuts of age 5, 10 or 15 years since clearing (8 replicas for each age class).
